# Secure Many-to-One Communications in Wireless Sensor Networks

**DOI:** 10.3390/s90705324

**Published:** 2009-07-07

**Authors:** Alexandre Viejo, Josep Domingo-Ferrer, Francesc Sebé, Jordi Castellà-Roca

**Affiliations:** 1 Department of Computer Engineering and Mathematics, UNESCO Chair in Data Privacy, Universitat Rovira i Virgili, Av. Països Catalans 26, E-43007 Tarragona, Spain; E-Mails: josep.domingo@urv.cat (J.D.-F.); jordi.castella@urv.cat (J.C.-R.); 2 Department of Mathematics, Universitat de Lleida, Av. Jaume II, 69, E-25001 Lleida, Spain; E-Mail: fsebe@matematica.udl.cat

**Keywords:** many-to-one communications, security, sensors, WSN

## Abstract

Wireless Sensor Networks (WSN) are formed by nodes with limited computational and power resources. WSNs are finding an increasing number of applications, both civilian and military, most of which require security for the sensed data being collected by the base station from remote sensor nodes. In addition, when many sensor nodes transmit to the base station, the implosion problem arises. Providing security measures and implosion-resistance in a resource-limited environment is a real challenge. This article reviews the aggregation strategies proposed in the literature to handle the bandwidth and security problems related to many-to-one transmission in WSNs. Recent contributions to secure lossless many-to-one communication developed by the authors in the context of several Spanish-funded projects are surveyed. Ongoing work on the secure lossy many-to-one communication is also sketched.

## 1. Introduction

A wireless sensor network (WSN) consists of resource-constrained devices equipped with wireless communication technologies and capable of sensing certain events from their environment. These devices are called *sensor nodes* and they are small and light (e.g., the size of a MICAz™ [1] mote is 58 mm *×* 32 mm *×* 7 mm and its weight is 3 g).

WSNs have a broad variety of applications for both civilian and military use. On the civilian side, this technology can be deployed to perform surveillance tasks, to provide emergency response in case of abnormal conditions in a residential or industrial area, to routinely monitor temperatures and pressures at several places in a large chemical plant, etc. On the military side, WSNs are attractive for their ease of deployment (especially in a hostile scenario, like a battlefield). In this kind of scenario, hundreds of sensor nodes are scattered around the area to be monitored. When this process ends, the sensor nodes use their wireless capabilities to establish connections and form the WSN. Finally, the whole network starts its activity [2].

Communications can be classified according to the number of involved senders and receivers. Single-sender paradigms are: *one-to-one* (unicast) in which a single sender transmits data to a single receiver; *one-to-all* (broadcast) in which one source sends data to all nodes of a network; and *one-to-many* (multicast) where a single source transmits to a given subset of nodes.

**Figure 1.**
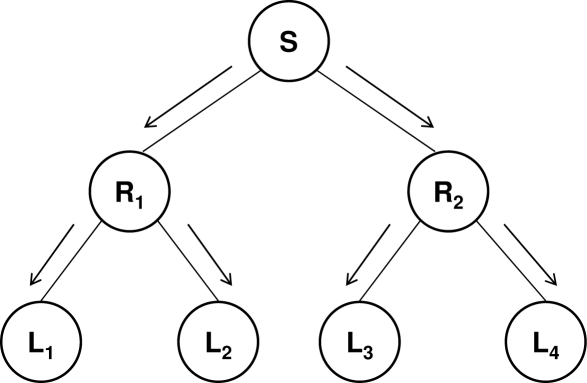
One-to-many communication using a tree topology.

Efficient one-to-many (and one-to-all) communications can be implemented using a tree topology (Figure 1). The root (*S*) of the tree is the source sending the data, the intermediate nodes are the routers (*R*_1_ and *R*_2_) that receive the content from their parent node and retransmit it to their child nodes (by replicating it for each child), and the leaves (*L*_1_, . . ., *L*_4_) are the receivers.

This model provides scalability because the number of receivers can be increased without increasing the workload or the bandwidth needs at the source. Unfortunately, a common monitoring application in WSN cannot be accommodated in this communication-efficient paradigm. This kind of application generally involves a large amount of sensor nodes sending sensed data towards a base station (BS) that processes all received information. This situation results in *many-to-one* communication [3]. If the number of transmitting nodes is large, the receiver may be overwhelmed by the incoming traffic. This problem is known as *implosion* [4].

**Figure 2.**
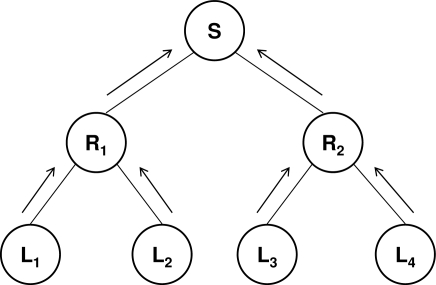
Many-to-one communication.

Implosion resistance is a challenging issue in the design of many-to-one communication protocols. These protocols also follow a tree topology. In this case, the leaves are the senders, the intermediate nodes are routers that collect the messages coming from their children and aggregate them into a single message that is transmitted upward to their parent, and the root (which corresponds to the BS of a WSN) is the only receiver (Figure 2). Scalability depends on the aggregation operation performed by the intermediate routers.

In addition to their scalability, many-to-one communications usually need to be secure. This is especially important in the case of WSNs. Let us assume that a certain entity is using a WSN to obtain some benefit (e.g., economic or military gain). If the network is deployed in an area that is very large and/or cannot be physically protected, its operation might be disrupted by an attacker having interests opposed to those of the owner of the WSN. The above is not a far-fetched scenario: WSNs are often deployed in disseminated and uncontrolled environments and, at the same time, their correct operation is often security-critical (e.g., emergency responses may depend on the collected sensor data). Therefore, in order to be really useful, communication protocols in WSNs should be designed by taking into account certain security requirements. These requirements should include *confidentiality* (an intruder should not be able to learn the transmitted data), *integrity* (any data alteration should be detectable by the receiver) and *authentication* (the source of the data should be verifiable by the receiver).

Due to the relevance and the usefulness of WSNs, the research community has focused on studying the security challenges that arise in the many-to-one communications in these networks. As a result, some proposals have been developed and some new and promising approaches can be found in the literature.

### 1.1. Cryptography for Lightweight Sensors

There is a general consensus that, in scenarios where nodes have limited computational capacity and power supply, the high cost of public-key cryptography is usually not affordable. Researchers assume that in such scenarios symmetric cryptography and hash functions constitute the basic tools to provide security.

However, [5] shows that public-key technology can still be selectively deployed in those environments. More specifically, the author argues that both the RSA [6] public-key cryptosystem with a small public exponent and the Rabin’s [7] public-key cryptosystem have fast algorithms for encryption and digital signature verification, and can be used on resource-constrained devices. Nevertheless, their decryption and signature generation are slow and resource-demanding, and therefore those operations cannot be used in WSNs.

Fortunately, the authors of [8] prove that elliptic curve cryptosystems (ECC) offer not only lightweight encryption and signature verification, but also lightweight decryption and signature generation suitable for WSNs.

More details on the feasibility of deploying public-key cryptography in WSNs can be found in [9], in which the author analyzes and puts in perspective some of the major research contributions of public-key cryptography in the WSN arena.

### 1.2. Contribution and Plan of This Paper

This paper identifies the challenges related to the secure many-to-one communications in WSNs and surveys the solutions proposed by these authors in the past years in the context of several Spanish-funded research projects. For those challenges which remain open, ongoing works that address them are described.

The rest of this paper is organized as follows. Section 2 reviews and classifies the aggregation strategies proposed in the literature to handle the bandwidth and security problems inherent to the many-to-one communication. Section 3 surveys our recent contributions to the secure lossless many-to-one communication. Ongoing work in the secure lossy many-to-one communication is described in Section 4. Conclusions are drawn in Section 5.

## 2. Many-to-One Data Transmission

The authors of [10] propose a solution for the implosion in many-to-one scenarios, in which intermediate routers combine received messages into a single message that is routed towards the BS. This process is called *aggregation*. More specifically, [10] presents a general framework for scalable many-to-one communication where intermediate nodes collect messages from their children, aggregate them and send a single aggregated message upward to their parent. In this way, the base station receives a single message containing readings from all sensor nodes. This solution allows an unlimited number of senders (it is scalable) as long as aggregated data do not grow in size. Two scenarios are then possible:

- *Lossy aggregation*. In this case, the message output by aggregation contains less information than the set of messages input to aggregation. Thus, the size of the output can stay the same as the size of each input. Some examples of lossy aggregation are:
  – If the data is a temperature, different temperatures can be aggregated by computing their average. Information loss comes from the fact that the base station will not learn the temperature obtained by each node but only the average of all readings.
  – If the data is a counter, different counters can be aggregated by addition. Information loss comes from the base station not being able to find out the exact contribution by each node.
  – If the data sent is a binary value indicating an alarm, it can be aggregated using a logical OR operation. The base station will know an alarm has been raised but not its exact origin. Lossy approaches cannot be used in scenarios where the BS must know the specific data sent by each sensor.
- *Lossless aggregation*. This situation occurs when no information loss is affordable during aggregation. It happens in applications where the BS multicasts a data request to the sensor nodes and these nodes react by sending one *q*-ary symbol each (data sent by each sensor can be modeled as an integer ranging from 1 to *q*). At the end of the process, the BS knows which symbol was transmitted by each sensor. In this case, the only possibility left is for sensor nodes to use a message length such that all the information they transmit can be aggregated into a single message of that length (the message reaching the BS). This implies that the actual informational content transmitted by sensor nodes will be less than the bitlength of the messages they use.

The framework presented in [10] works fine with both types of aggregation mentioned above. However, it does not address security issues. This fact represents a major drawback which disqualifies it when security requirements arise. As argued in the introduction above, the lack of security in WSNs can jeopardize their practical applicability. Accordingly, we will focus on security-aware solutions.

In order to provide some background, we next review the most relevant schemes in the literature for *secure many-to-one lossless transmission* and *secure many-to-one lossy transmission*.

### 2.1. Secure Many-to-One Lossless Transmission

Proposals that focus on this scenario can be divided in two categories described below: *secure acknowledgment* and *secure symbol transmission*.

#### Secure Acknowledgment

These schemes provide the BS with an undeniable and unforgeable proof that a certain set of leaves have received a specific content. The information sent by the leaves to the BS is unary in the sense that, after receiving a piece of data, every sensor will either respond with a positive acknowledgment (a digital signature) in case of correct reception or stay silent if otherwise.

The systems proposed in [11] and [12] fall into this category. The former uses the multisignature scheme in [13] constructed over a Gap Diffie-Hellman group (GDH) [14]. The latter is a construction whose security rests on the hardness of the discrete logarithm problem. Both solutions provide non-repudiation and are scalable (*O*(1) message length) as long as the set of acknowledging leaves remains stable.

These systems only provide non-repudiation; other security properties are not addressed. For instance, the BS is unable to distinguish a voluntary non-transmission from malicious erasure of acknowledgments by intruders. The authors in [11] and [12] leave this issue for future work. Thus, integrity is not ensured. Confidentiality is not achieved either since any intruder listening to the communication can ascertain which leaves are acknowledging and which are not.

### Secure Symbol Transmission

Here, the BS first multicasts a data request to the sensor nodes. Then, upon reception of this request, the sensors react by sending one *q*-ary symbol each. These messages will be aggregated by the intermediate nodes. From the received aggregate message, the BS can obtain the symbol sent by each sensor.

It is easy to prove that symbols sent by *n* nodes cannot be aggregated in a message whose length is less than *O*(*n*) when all symbols have the same probability of being sent. According to that, research in this scenario focuses on designing systems whose actual message length is as short as possible (within the *O*(*n*) length class). Note that this does not permit an unlimited amount of senders.

In [15], a system based on super-increasing sequences and additive privacy homomorphisms is described. The length of messages is *O*(*n*), where *n* is the number of leaves of the multicast tree. If implemented using the Okamoto-Uchiyama cryptosystem [16] for binary transmissions, the message length asymptotically tends to 6*n*. The scheme can be easily extended to accommodate *q*-ary alphabets with message length tending to 3*tn*, where *t* is the smallest integer such that *q* ≤ 2*^t^* − 1.

The scheme in [17] reduces the message length with respect to the scheme presented in [15] for the case of biased *binary* communication, i.e., when the probability of leaves transmitting a ‘1’ symbol is less than the probability of their transmitting a ‘0’ symbol. This scheme offers an *O*(*k* log *k* log *n*) message length with *n* being the number of leaves and *k* being an upper bound on the number of leaves that wish to simultaneously transmit the least likely symbol.

Both systems mentioned in this section provide confidentiality, authentication and integrity, and they use bandwidth efficiently. Non-repudiation is not provided by any of them, but their main drawback is the high computational cost. Both use additive public-key privacy homomorphisms, whose clear-text message length grows like *O*(*n*) for [15] and *O*(*k* log *k* log *n*) for [17]. The costly cryptographic operations on long messages required by these schemes render them ill-suited for implementation on resource-limited hardware like the sensor nodes used in WSNs.

Regarding integrity, both systems permit data corruption to be detected, but the identification of the corrupting nodes is not straightforward and must be done using a tracing procedure described in [17] (also applicable to [15]), through which the BS traces and identifies corrupting nodes.

### 2.2. Secure Many-to-One Lossy Transmission

Schemes that fall in this category focus on obtaining aggregate statistics such as SUM, AVERAGE, or MAX/MIN of data readings over a certain space region or time period. These protocols provide different levels of security to the data sent by the sensors. The computation and communication resources needed must be also considered.

Two different approaches can be distinguished: *cleartext data aggregation* and *encrypted data aggregation*.

#### Cleartext Data Aggregation

In this class of schemes, the nodes that perform the aggregation can read the data they are aggregating and can compute any aggregation function on them. Therefore, if the messages are encrypted, the intermediate nodes must be able to decrypt them. The problem is that, when an intermediate node is compromised, the confidentiality and the integrity of all messages traversing that node also become compromised.

[18–20] are similar protocols that use symmetric cryptography for link encryption to offer protection only against external attackers who eavesdrop transmitted messages.

The authors of [21] introduce a protocol that does not use any kind of encryption, so that no confidentiality is offered against either external or internal attackers. Nevertheless, this proposal achieves integrity against compromised nodes. As a drawback, integrity is only guaranteed if there are no two consecutive compromised nodes (the parent node and its child) in the path towards the BS.

Another scheme that only addresses integrity can be found in [22]. This proposal is a special case in this category because it only covers one particular aggregation function. More specifically, the authors introduce a solution for computing the median of inputs in the presence of a fraction of compromised nodes in lightweight WSNs. The main problem of this contribution is its lack of confidentiality against external or internal adversaries.

[23] improves [21] by providing integrity in scenarios with two or more consecutive compromised nodes. This protocol uses symmetric encryption to offer confidentiality only against external users. In addition, as proven in [24], this scheme uses a large amount of bandwidth to ensure a reasonable level of security. This is an important drawback in resource-constrained environments like WSNs.

#### Encrypted Data Aggregation

All proposals in this class aggregate encrypted data directly. Data is not decrypted at intermediate nodes, hence the confidentiality of the data traversing the network towards the BS is preserved.

Unfortunately, confidentiality is not provided without cost: schemes in this class allow only one function (or at best a restricted set of functions) to be computed by intermediate nodes on the encrypted data sent by the leaves.

Jadia *et al.* introduce in [25] an enhanced version of [21] that offers confidentiality against external and internal attackers. This upgrade is based on the use of a symmetric-key cryptosystem where encryption is performed by adding to the data a sufficiently long random encryption key [26]. This system allows data aggregation by means of addition. The main shortcoming of this proposal is its weakness against two consecutive compromised nodes (a problem inherited from [21]). If this situation occurs, integrity is no longer guaranteed.

[27] proposes a new protocol based on additive homomorphic encryption. The goal of [27] is to prevent a passive attacker (eavesdropper) from gaining any information about the sensor data. Thanks to homomorphic encryption, intermediate nodes can aggregate encrypted data directly. However, this scheme has some shortcomings:

- Integrity is not provided (although this is addressed in [28]).
- The use of a homomorphic cryptosystem is ill-suited to resource-constrained environments.
- This protocol generates significant overhead if the network is unreliable, since the identities of non-responding sensor nodes must be sent to the BS together with the aggregated result.

In [29], another scheme based on homomorphic encryption is presented. The authors show how relevant aggregation functions can be constructed with an additive privacy homomorphism. More specifically, they illustrate their approach with the aggregation functions “average” and “movement detection”. They also provide a key pre-distribution scheme that suits end-to-end encryption of many-to-one traffic in WSNs. However, this scheme has some drawbacks as well: it suffers from a high computational cost and integrity is not achieved.

Sun *et al.* present in [30] a protocol combining homomorphic encryption with an aggregate signature scheme. As a result, this scheme provides confidentiality and integrity. [31] is a similar approach where confidentiality is achieved using additive homomorphic encryption and integrity is guaranteed using peer nodes that monitor the behavior of computing peers. Unfortunately, both schemes use costly cryptographic operations that cannot be used in resource-constrained environments.

Authors in [32] present two protocols for additive aggregation functions that preserve the confidentiality of the data. Both schemes are lightweight enough to work properly in WSNs. However, they suffer from significant communication overhead due to message exchange between the nodes of the network (which are organized in clusters). One additional drawback of the first protocol is the computational overhead of data aggregation within those clusters of nodes. The second protocol reduces computational overhead at the cost of increasing bandwidth consumption. Studies such as [33] and [34] show that data transmission consumes much more energy than computation. Thus, the high communication overhead incurred by both schemes limits the energy lifetime of the network and consequently its functionality.

## 3. Our Recent Contributions to Secure Lossless Communication

In Section 2.1 we pointed out the need to design efficient protocols to provide secure many-to-one symbol transmission for WSNs. We next survey in some detail our recent contributions to this field.

As mentioned above, sensors are devices with limited computational capabilities and limited battery power. Therefore, WSNs require protocols which *use bandwidth sparingly* and *require little computation by the sensors*.

In [35] we introduce a scheme for the many-to-one secure symbol transmission that achieves minimum message length and thus minimizes bandwidth consumption. This proposal is useful in environments where the bandwidth is a scarce resource and it is critical to make the most of it. The system also offers immediate detection of corrupted messages. The underlying cryptography consists of multisignatures over Gap Diffie-Hellman (GDH) groups [13]. Note that these cryptographic operations may not be suitable for implementation in WSNs.

We solve this situation in [36]. This is the first protocol in the literature that offers secure lossless many-to-one symbol transmission for WSNs. This scheme also achieves minimum message length (the same achieved by [35]), but replaces the use of GDH cryptography with hash functions. In this way, the computational cost at nodes is reduced. As a result, this proposal is suitable for resource-constrained devices like the ones used in WSNs. The tradeoff is that this scheme does not permit immediate detection of corrupted messages. Such a detection can be performed using an *a posteriori* tracing algorithm, which is more efficient than the tracing procedure presented in [17].

We next explain how this novel protocol works. Further details can be found in [36].

### 3.1. Protocol for Secure Many-to-One Symbol Transmission in WSNs

#### General Assumptions

The BS is a full-fledged computer. Sensor nodes and intermediate nodes are low-cost devices. The BS owns a private key *SK_BS_*. The corresponding public key *PK_BS_* is known and accepted as valid by all nodes in the network. Let *n* be the number of sensors and *U_i_*, 1 ≤ *i* ≤ *n*, denote the sensor nodes. Each sensor *U_i_* shares a secret key *K_i_* with the BS.

#### Many-to-One *q*-Ary Transmission

We represent each symbol from the *q*-ary alphabet by a different integer from the set {1, . . ., *q*}. Parameter *t* is chosen as the smallest integer satisfying *q* ≤ 2*^t^ −* 1. Parameter *s* is a security parameter. A protocol execution consists of the following steps:

1. *Challenge*. The BS generates a random value *v* and signs it to obtain {*v*}*_SK_BS__* (the signature algorithm selected should be such that the computational cost of signature verification is low). The signed value is multicast by the BS to all sensors.
2. *Message generation.*
  - Upon receiving *v* and verifying its signature, each sensor *U_i_* computes a pseudo-random *t*-bit sequence (*c*_1_, . . ., *c_t_*) ← *lsb_t_*(*𝒣*(*v||K_i_*)), where *lsb_t_*(*·*) is a function returning the *t* least significant bits of its argument, *𝒣* is a one-way hash function and || is the concatenation operator.
  - Each *U_i_* computes a sequence (*d*_1_, . . ., *d_t_*) as follows. Let (*b*_1_, . . ., *b_t_*) be the binary representation of the *q*-ary symbol to be transmitted by *U_i_*.
    - If (*b*_1_, . . ., *b_t_*) = (*c*_1_, . . ., *c_t_*) then (*d*_1_, . . ., *d_t_*) = (*b*_1_, . . ., *b_t_*)
      Else (*d*_1_, . . ., *d_t_*) = (*b*_1_ *⊗ c*_1_, . . ., *b_t_ ⊗ c_t_*)
    Note that this step ensures that the sequence (*d*_1_, . . ., *d_t_*) does not have all of its elements equal to 0. This all zero value is reserved to identify non-transmittal by sensor nodes.
  - *U_i_* computes an *s*-bit pseudo-random integer *σ_i_* as follows:
    $$\sigma_{i}\leftarrow {\mathrm{lsb}}_{s}\;\left(𝒣\left(d_{1},\ldots ,\;d_{t}\left\|v\right\|K_{i}\right)\right)$$
  - Each *U_i_* generates a *tn*-bit sequence (*n* is the number of leaves) *I_i_* and sets the bits from the subsequence between positions *t*(*i −* 1) + 1 and *ti* so that they match (*d*_1_, . . ., *d_t_*). The remaining bits are set to “0”.
  - *U_i_* sends the pair (*I_i_, σ_i_*) up to its parent node.
3. *Message aggregation*. An intermediate node *R* (or the BS) receives messages from its child routers/sensor nodes and does the following:
  - Store each received pair (*I_j_, σ_j_*) (they may have to be checked later).
  - Once all expected messages {(*I_j_*, *σ_j_*)}*_j_* have been received, aggregate them by computing *I* = V*_j_ I_j_* (∨ denotes the bitwise OR operation) and σ = ∑_*j*_ σ_*j*_ (mod 2^*s*^).
  - If *R* is not the BS, send (*I, σ*) upward to its parent node. Otherwise, this is the final aggregated message.
4. *Symbol extraction*. From the final aggregated message (*I, σ*), the BS obtains, for each sensor *U_i_*, the binary representation (*b_i,_*_1_, . . ., *b_i,t_*) of the symbol sent by the leaf. It is obtained from the sequence (*d_i,_*_1_, . . ., *d_i,t_*), previously generated by *U_i_* (see Step 2b), which is contained in *I*. Then the BS computes the pseudo-random integer linked to (*d_i,_*_1_, . . ., *d_i,t_*) (see Step 2c), which will be used to check the integrity of the whole aggregated message. We next give the pseudo-code related to this process:
  - Let *i* = 1, *ω* = 0.
  - While *i ≤ n* loop
    - Compute (*c*_1_, . . ., *c_t_*) ← *lsb_t_*(*𝒣*(*v*||*K_i_*)).
    - If (*I*[*t*(*i* − 1) + 1], . . ., *I*[*ti*]) = (*c*_1_, . . ., *c_t_*) then (*b*_i,1_, . . ., *b_i,t_*) = (*c*_1_, . . ., *c_t_*).
    - Else (*b_i_*_,1_, . . ., *b_i,t_*) = (*I*[*t*(*i* − 1) + 1] ⊗ *c*_1_, . . ., *I*[*ti*] ⊗ *c_t_*).
    - Compute *ϕ_i_* ← *lsb_s_*(*𝒣*(*I*[*t*(*i* − 1) + 1], . . ., *I*[*ti*]||*v*||*K_i_*)).
    - *ω* = *ω* + *ϕ_i_* (mod 2*^s^*).
    - *i* = *i* + 1.
  - If *ω* = *σ* then return *B* = ((*b*_1_*_,_*_1_, . . ., *b*_1_*_,t_*), . . . , (*b_n,_*_1_, . . ., *b_n,t_*)), where (*b_i,_*_1_, . . ., *b_i,t_*) is the binary representation of the symbol transmitted by *U_i_*. The BS also multicasts a signed acknowledgment {“*Ack*”*||v*}*_SK_BS__* to the sensor nodes. This message contains the challenge *v* to avoid replay attacks. Upon receiving this message, intermediate routers remove messages stored at Step 3a.
    If *≠* = *σ*, the BS launches the error-tracing procedure explained in the next section.

**Figure 3.**
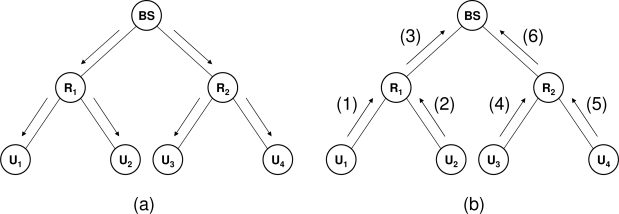
Message flow in a protocol execution.

Figure 3 shows the message flow generated by a protocol execution in a simple scenario with one base station (*BS*), two intermediate nodes (*R*_1_ and *R*_2_) and four sensors (*U*_1_, . . ., *U*_4_). In Figure 3a the BS broadcasts a challenge to all sensor nodes (Step 1 in the protocol execution). In Figure 3b, message (1) sent by *U*_1_ corresponds to the pair (*I*_1_*, σ*_1_) while message (2) sent by *U*_2_ represents the pair (*I*_2_*, σ*_2_) (Step 2 in the protocol execution). Node *R*_1_ constructs message (3), which corresponds to (*I, σ*), by aggregating messages (1) and (2) (Step 3 in the protocol execution). The same process occurs in the subtree rooted by *R*_2_. The latter node constructs message (6) by aggregating messages (4) and (5), which correspond to the pairs (*I*_3_*, σ*_3_) and (*I*_4_*, σ*_4_), respectively. Eventually, the BS aggregates messages (3) and (6) to get the final aggregated message. After that, the BS extracts the symbols transmitted by the sensor nodes (Step 4 in the protocol execution).

#### Procedure to Deal with Corrupted Messages

During symbol extraction, the BS checks the integrity of the received message. If this verification fails, the BS identifies the message as corrupted.

For the received corrupted aggregate *I* component, BS computes the valid *σ_i_* associated to each *U_i_* (using the shared key *K_i_*) and sends to all nodes the signed message

$${\left\{I\left\|\sigma_{1},\ldots ,\sigma_{n}\right\|v\right\}}_{{\mathrm{SK}}_{\mathrm{BS}}}$$

Each intermediate *R* node verifies its signature *σ* and the challenge *v* and checks each stored message (*I_j_, σ_j_*) received from its children. In this way, corrupting children can be detected (and removed). The detailed procedure is described in [36].

#### Message Length Optimization

Our system is designed for nodes with limited computational capacity and power supply. This motivates the need to reduce energy consumption as much as possible. Reducing the length of messages is one way to achieve this.

In our protocol, sensor *U_i_* sends (*I_i_, σ_i_*) where *I_i_* is a *tn*-bit long binary sequence. Useful information within *I_i_* is contained in bits located between positions *t*(*i −* 1) + 1 and *ti*. The remaining bits of *I_i_* are set to 0.

This information could be represented in a more compact way using log *n* bits to encode index *i* and *t* bits for useful information. In this way, the length of *I_i_* would be *t* + log *n*. Aggregation of vectors *I_i_* would be done by concatenation. In this way, the length of a vector *I* containing data from *j* leaves would be *j*(*t* + log *n*) bits.

For small values of *j* this results in shorter messages than those described in our protocol above (i.e. when *j*(*t* + log *n*) *< tn*). Low values of *j* appear at nodes that are far from the BS. However, when *j* grows towards *n* this new coding results in longer messages than those described above.

Therefore using this alternative coding when *j* satisfies *j*(*t* + log *n*) *< tn* (near the leaves) and switching to the initial coding when messages get near the root is a way to minimize the length of transmitted data.

## 4. Open Issues and Work in Progress on Secure Lossy Communications

We have argued in Section 2.2 that new lightweight protocols providing secure many-to-one lossy transmission are needed. These schemes must allow the BS to obtain aggregate statistics such as SUM, AVERAGE, or MAX/MIN of the data gathered by the sensor nodes. Those proposals should guarantee confidentiality and integrity.

We are currently working in two different contributions in this direction. Both schemes are at an advanced stage of development and we next introduce their main features.

### 4.1. MAX, MIN and Range Queries

The first scheme is a probabilistic protocol that allows the BS to query the WSN in order to obtain the MAX of the sensed data. Our scenario requires sensor nodes to be capable of computing hash functions and intermediate nodes to be capable of computing bitwise operations. This protocol will prevent an adversary from learning the reading sent by each sensor node (confidentiality against external and internal attackers will be achieved). Besides, we will show that the proposed protocol is also resilient against an adversary who actively tries to alter the readings sent by the sensor nodes, thereby providing data integrity. The proposed solution will be efficient in terms of both the computation and communication required; moreover, the communication overhead will be evenly distributed among nodes, hence increasing the power lifetime of the WSN.

It is straightforward to extend this first protocol to compute the MIN, as well as to perform range queries (i.e., to query whether there is any node that has sensed values within a given range). In turn, range queries are a building block for more complex queries.

### 4.2. General Queries

In a second protocol in preparation, we propose mixing both lossless and lossy approaches to obtain a secure and scalable protocol designed to provide secure many-to-one *lossy* transmission of *q*-ary symbols. As a main feature, this novel protocol will allow the BS to compute any mathematical function (average, variance, minimum, maximum, etc.) on the data sent by sensor nodes.

In the initial version of the protocol, confidentiality will be guaranteed by having intermediate nodes aggregate encrypted data directly. The required process may be excessively resource-demanding and thus not suitable for lightweight WSNs. This will be improved in a subsequent version of the system. Perfect integrity will be achieved only against individual dishonest nodes trying to alter transmitted data. Colluding nodes may modify the final result of a mathematical function applied to the received data, but they will be able to cause only a limited deviation from the correct result. Finally, authentication will be provided in the sense that the BS will be able to verify that all data come from authorized sensors.

Regarding the transmission cost of the protocol, we plan to offer a message length growing logarithmically with the number of sensors of the network, which guarantees scalability.

## 5. Conclusions

Due to their resource-constrained nature, their security-critical application and the implosion problems caused by their many-to-one traffic, wireless sensor networks are a challenging technology.

We have reviewed and classified the aggregation strategies used to merge the information sent by a community of sensor nodes into a single message to be collected by a base station. Some of these strategies fail to provide security and others provide security with a computation cost unaffordable by WSN nodes. Recent contributions to the secure lossless many-to-one communication developed by these authors in the context of several Spanish-funded research projects have been reviewed.

Lossy many-to-one communication is an attractive option when the bandwidth problem is the main concern. To guarantee security in lossy scenarios with reasonable computational burden is an open issue. We have sketched work in progress in this direction.
